# First Complete Mitochondrial Genome Analysis of Tree Frog, *Dryophytes flaviventris* and Comparison with *Dryophytes suweonensis*

**DOI:** 10.3390/ijms26062423

**Published:** 2025-03-07

**Authors:** Nakyung Yoo, Kang-Rae Kim, Biet Thanh Tran, Keun-Yong Kim, Mi-Sook Min, Ju-Duk Yoon, Keun-Sik Kim

**Affiliations:** 1 Restoration Research Team (Fishes/Amphibians & Reptile), Research Center for Endangered Species, National Institute of Ecology, Yeongyang 36531, Republic of Korea; nkyoo96@nie.re.kr; 2 Southeast Sea Fisheries Research Institute, National Institute of Fisheries Science, Namhae 53085, Republic of Korea; kimkangrae9586@gmail.com; 3 Genetic Analysis Team, AquaGenTech Co., Ltd., Busan 48228, Republic of Korea; aquagentech@naver.com (B.T.T.); koby0323@hanmail.net (K.-Y.K.); 4 Conservation Genome Resource Bank for Korean Wildlife (CGRB), BK21 Program for Veterinary Science, College of Veterinary Medicine, Seoul National University, Seoul 08826, Republic of Korea; minbio@yahoo.co.kr

**Keywords:** tree frog, mitogenome, *Dryophytes flaviventris*, *Dryophytes suweonensis*, phylogeny, taxonomy

## Abstract

Mitochondrial genomes (mitogenomes) play a key role in species identification and phylogenetic studies due to their stable gene arrangements and evolutionary insights. *Dryophytes flaviventris*, classified in 2020 and closely related to *D. suweonensis*, lacks mitochondrial DNA data for differentiation. This gap hinders accurate species identification, highlighting the need for further genomic studies. The complete mitogenome size of two *D. flaviventris* were 18,616–18,617 bp and those for two *D. suweonensis* were 18,610–18,616 bp, the mitogenomes of the two species consisting of 13 protein-coding genes (PCGs), two ribosomal RNA genes, 22 transfer RNA (tRNA) genes, and a D-loop. Phylogenetic analysis confirmed that the mitochondrial DNA of all four individuals formed a monophyletic group, showing no genetic differentiation. As a result, the two species do not form distinct clades, and mitogenomes could not differentiate them, suggesting they are not reciprocally monophyletic. This study presents the first mitogenome data for *D. flaviventris* and provides valuable insights into tree frog taxonomy.

## 1. Introduction

Mitochondrial DNA (mtDNA) is maternally inherited and non-recombining, making it suitable for tracing the evolutionary history of specific lineages [1]. MtDNA is also valuable for estimating divergence times across various taxa, with estimates reaching as far back as 300 million years [2,3].

The mtDNA of vertebrates is typically small, averaging around 16 kb, which facilitates whole-genome sequencing [4]. While gene arrangements in mtDNA tend to be stable within major groups [4], variations do occur, and comparing these arrangements can reveal deep branches in the phylogenetic tree of multicellular animals [4,5]. This stability makes mtDNA valuable for inferring phylogenetic relationships across species. Additionally, mtDNA exists in multiple copies within cells, making extraction and analysis more efficient [1]. This feature is especially advantageous when working with endangered wildlife or degraded samples. Furthermore, the faster evolutionary rate of mtDNA compared to nuclear DNA makes it well suited to detect subtle genetic differences between closely related species [2].

In the case of frogs, morphological characteristics alone have limitations in distinguishing species, making genetic markers the most accurate method for species identification [6,7]. The mitochondrial genome (mitogenome), with its suitable size and stability, provides robust data for investigating the phylogenetics of anurans [8]. Given that most family-level taxa are represented by a single species, analyzing mitogenome sequences in anurans is particularly useful for confirming phylogenetic relationships.

According to Duellman et al. (2016) [9], two Nearctic hylid clades are distinctly clustered in phylogenetic trees. One genus, *Hyla*, is endemic to the Old World, while the other, *Dryophytes*, is primarily distributed in the New World, with three species found in Asia. *Dryophytes* species are typically medium-sized, arboreal frogs with green coloration and toe pads. Although they are closely related to the genus *Hyla*, they do not exhibit significant morphological differences from *Hyla*. The genus *Dryophytes* includes 19 species, of which, 16 species are endemic to eastern North America, and the other three are found in Asia (Korea, China, Japan, and Russia).

Only two tree frogs species were recognized in the Republic of Korea before 2020: the Suweon tree frog (*Dryophytes suweonensis*) and the Japanese tree frog (*D. japonicus*), which was classified under *Hyla* until 2016 [9]. However, with the recent identification of *Dryophytes flaviventris*, the Republic of Korea now has three tree frog species. Borzée et al. (2020) [10] reported the discovery of *D. flaviventris*, which is geographically segregated from *D. suweonensis* by the Chilgap mountain range. While *D. suweonensis* inhabits large plains along the western coast of the Republic of Korea, *D. flaviventris* has a more restricted range and may face greater threats. Given that *D. suweonensis* is already classified as endangered (EN) on the IUCN Red List [11], there is an urgent need for further research to definitively differentiate *D. flaviventris* from *D. suweonensis* and assess the conservation status of *D. flaviventris*, which may also warrant its classification as an endangered species. Additionally, *D. flaviventris* is morphologically distinguishable from *D. suweonensis* by a smaller web between its second and third toes. Borzée et al. (2020) [10] presented a phylogenetic tree using double-digest RAD sequencing (ddRAD) analysis to show genetic differences between the two species. However, their study did not include mtDNA analysis, which could more clearly distinguish the two species.

This study presents the first complete analysis of the mitogenome of *D. flaviventris*. To establish genetic information for the tree frog species in the Republic of Korea and to identify endangered species such as *D. flaviventris* and *D. suweonensis*, it is necessary to analyze and compare their complete mitogenomes. This is the inaugural comprehensive analysis of the mitogenome of *D. flaviventris*, which will provide critical data for future taxonomic and conservation studies.

## 2. Results and Discussion

### 2.1. Basic Characteristics of D. flaviventris Mitogenomes

The complete mitochondrial genomes of *D. flaviventris* (Dfl03 and Dfl04) and *D. suweonensis* (Dsu03 and Dsu04) were sequenced using long-range PCR and iSeq100 sequencing (Supplementary Table S1). The sequencing quality was high, with Q20 (>97%) and Q30 (>93%) scores. Mapping to the reference genome (GenBank accession number KY419887) showed 100% coverage, with mean depths ranging from 3771× to 5382× (Supplementary Table S2). Coverage depth analysis revealed variations across the mitochondrial genome, with regions of higher read accumulation (Supplementary Figure S1).

**Figure 1:**
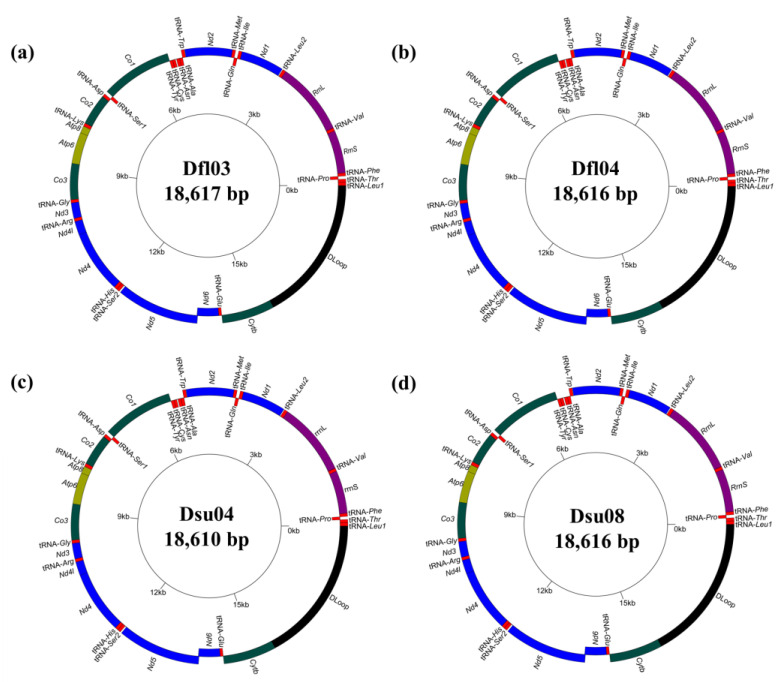
Circular maps of the mitogenomes of the two tree frogs, *Dryophytes flaviventris* Df103 (**a**) and Dfl04 (**b**) and *Dryophytes suweonensis* Dsu04 (**c**) and Dsu08 (**d**).

The complete and circular mitogenome sequences of *D. flaviventris* ranged from 18,616 to 18,617 bp in size (Figure 1). With few exceptions, the mitogenomes of most animals have a consistent gene structure, comprising 13 PCGs, two rRNAs, 22 tRNAs, and the non-coding region [4]. The mitogenomes of the two specimens of *D. flaviventris* used for comparison also contained 37 mitochondrial genes, including 13 PCGs, two rRNA genes, 22 tRNA genes, and the non-coding control region. The majority strand (H-strand) contained 12 PCGs, 14 tRNAs, and two rRNAs, and the minority strand (L-strand) contained one PCG and eight tRNAs. The base compositions were as follows: A (29.12–29.13%), T (28.48–28.50%), C (27.44–27.47%), G (14.92–14.93%), and G + C contents ranged from 42.36–42.40%. The analysis of the complete mitogenomes’ nucleotide skew showed that they had a negative GC-skew value (−0.30) but a positive AT-skew value (0.01).

We compared one *D. japonicus* (AB303949) and two *D. suweonensis* (DSU04 and DSU08) to the two *D. flaviventris* from the Republic of Korea. The complete and circular mitogenome sequences of *D. suweonensis* ranged from 18,610 to 18,616 bp, which differed from *D. flaviventris* by 0–7 bp (Figure 1), while the *D. japonicus* mitogenome comprised 19,519 bp. The GC-skew of the full-length mitogenomes among the three species varied from −0.30 to −0.27; however, the AT-skew varied from −0.02 to 0.01. These results show that, compared with other *Dryophytes* species, the whole mitogenomes of *D. suweonensis* and *D. flaviventris* have higher AT-skew values and the lowest GC-skew values. The overall G + C contents of *D. suweonensis* ranged from 42.37 to 42.39%, showing that there was little difference from the G + C content of *D. flaviventris*.

According to the findings of Frost and Darrel (2024), *D. suweonensis* and *D. immaculatus* are considered synonymous, and *D. japonicus* is regarded as the same species as *D. ussuriensis* [12]. In both cases, the identity rates reached mean that there is little difference in the size of the mitogenomes, with an identity rate reaching 99% [13]. In this study, the identity rate between all the specimens of *D. flaviventris* and *D. suweonensis* was also close to 99% (99.6–99.8%), and the mitogenome size differences between *D. flaviventris* and *D. suweonensis* were found to be less than 10 bp. Therefore, based on the mitogenome sequence similarity between *D. flaviventris* and *D. suweonensis*, we suggest that they are taxonomically and phylogenetically the same species.

### 2.2. PCGs and Codon Usage Patterns of D. flaviventris

**Figure 2:**
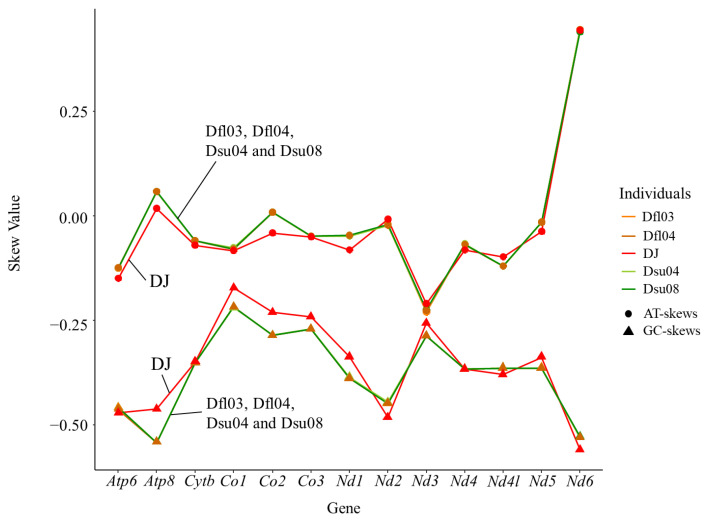
AT- and GC-skews of 13 protein-coding genes of mitogenomes of two *Dryophytes flaviventris* (Dfl03 and Dfl04) and *D. suweonensis* (Dsu04 and Dsu08) in this study and *D. japonica* (DJ; GenBank accession number AB303949). Three PCGs (*Nd1*, *Co2*, and *Nd3*) ended with incomplete stop codons, T. These may presumably be completed by post-transcriptional polyadenylation with a poly-A tail [14]. *D. japonicus* also had incomplete T stop codons at the same positions, and *D. immaculata*, which is phylogenetically close to *D. suweonensis*, had T at the same positions, but additionally exhibited incomplete TA stop codons in *Co3* [15,16].

The total length of the 13 PCGs was 11,304 bp. The A + T contents (56.48–56.52%) in the PCGs were higher than the G + C contents (43.48–43.52%). The AT-skew (−0.03) and the GC-skew (−0.35) were negative (Figure 2; Table 1). Among the 13 PCGs, three PCGs used ATT, nine PCGs used ATG, and two PCGs used GTG as their start codon. Four PCGs (*Nd2*, *Co1*, *Nd5*, and *Nd6*) used AGA as the stop codon, five PCGs (*Atp8*, *Atp6*, and *Co3*) used TAA, and *Cytb* used TAG.

The A + T contents of the 13 PCGs of *D. suweonensis* were 56.49–56.51%, the AT-skew values were −0.03, and the GC-skew values were −0.35 (Figure 2; Table 1).

**Figure 3:**
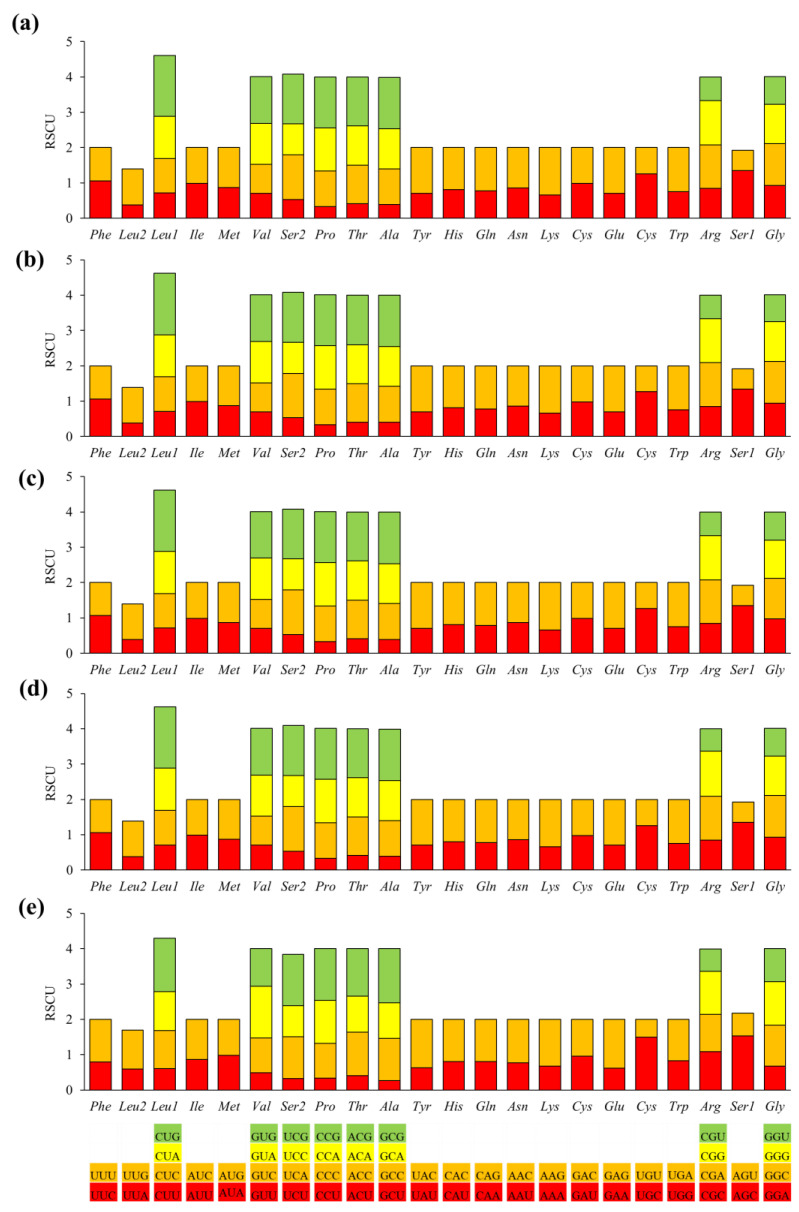
The relative synonymous codon usage (RSCU) of the 13 protein-coding genes in the mitogenomes of *Dryophytes flaviventris* Dfl03 (**a**) and Dfl04 (**b**), *D. suweonensis* Dsu04 (**c**) and Dsu08 (**d**), and *D. japonicus* (**e**). One of the codons ATT, ATC, ATA, ATG, or GTG typically serves as a mitochondrial start codon in most vertebrates. However, in the complete mitogenome sequences of *D. suweonensis* (KY419887 and NC_032380) deposited in GenBank database, the *Nd1* gene was reported to start with TTG (leucine). Similarly, in this study, the *Nd1* regions of two *Dryophytes* species were identified as starting with TTG (leucine). Most PCGs typically began with an ATG codon [17], except for *Nd1*, which started with ATT, and *Nd3*, which started with GTG.

Figure 3 shows the codon usage and relative synonymous codon usage (RSCU) values. The RSCU analysis shows clear differences between *D. japonicus* and *D. flaviventris* with *D. suweonensis*, while the RSCUs of *D. flaviventris* and *D. suweonensis* are an exact match. RSCU is often used to distinguish species, so, based on this, *D. japonicus* can be considered a different species from *D. flaviventris* and *D. suweonensis*, while their identical RSCU values suggest that *D. flaviventris* and *D. suweonensis* are likely conspecific or extremely closely related.

Nine of 64 codons showed the highest use frequencies, and they were CCU (145–146), CUU (144–145), CCC (124), AAU (118), CAU (111), AUU (108), AUC (105), UAU (105), and CCA (102). However, the GUG (18) and GCG (14) codons were the least used stop codons. Among them, the RSCU value of 34 codons was greater than 1 (1.01–1.74), indicating that these codons were used more frequently.

### 2.3. rRNA and tRNA Genes of D. flaviventris

In the case of both *D. flaviventris* mitogenomes, two rRNA genes (12S and 16S rRNA genes) were found to be encoded by the H-strand. The 12S rRNA gene was 934 bp in *D. flaviventris,* and it was located between the tRNA-*Phe* and tRNA-*Val* genes. The length of the 16S rRNA gene in *D. flaviventris* was found to be 1601 bp, situated between the tRNA-*Val* and tRNA-*Leu2* genes. The A + T content was higher than the G + C content, measuring at 58.8%. The AT-skew was slightly positive, whereas the GC-skew was strongly negative (Table 1).

A total of 22 tRNA genes were identified in *D. flaviventris* mitogenomes, similar to typical vertebrates. Of these, 14 tRNA genes were located on the H-strand and eight genes were located on the L-strand. All tRNAs, except for tRNA-*Ser*, had the typical dihydrouridine (DHU) arm. The lack of a DHU arm in tRNA-*Ser*, as suggested by Ohtsuki et al. (2002) [18], indicates that incomplete tRNA could still fit into the ribosome by adjusting its function and structural conformation.

### 2.4. Non-Coding Region of D. flaviventris

A putative control (D-loop) region of *D. flaviventris* with 3195–3196 bp was identified. It was located between the *Cytb* and the tRNA-*Leu1* gene on the H-strand (Figure 1). Its A + T contents of *D. flaviventris* were 59.98–60.13%. The AT/GC-skews showed that *D. flaviventris* has a positive AT-skew (0.012–0.018) and also a strongly negative GC-skew (−0.305–−0.300) (Table 1). The three *Dryophytes* species in the Republic of Korea ranged from 3,189 to 4,103 bp. Among of them, the A + T contents in the D-loop region were as follows: *D. suweonensis* 60.02–60.11% and *D. japonicus* 61.69%. AT/GC-skew analysis showed that the D-loop gene of *D. suweonensis*, like that of *D. flaviventris*, exhibited a positive AT-skew (0.012–0.016), whereas it was negative in *D. japonicus* (−0.074).

### 2.5. Repeat Detection in D. flaviventris

**Figure 4:**
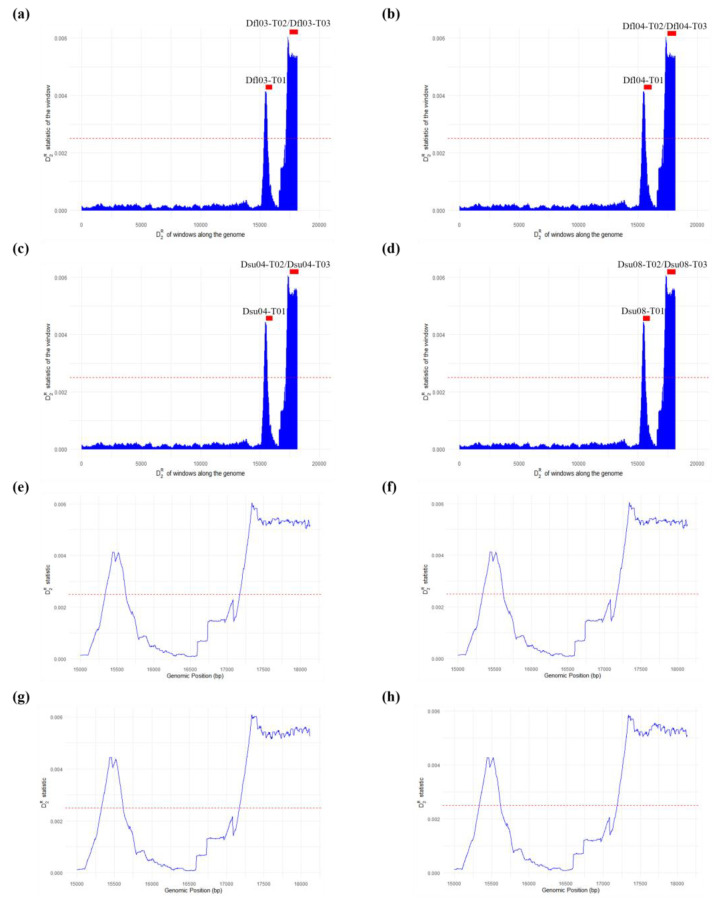
Repeat detection in the mitogenomes of *Dryophytes flaviventris* and *D. suweonensis* based on $D_{2}^{R}$ values. (**a**–**d**) The distribution of $D_{2}^{R}$ values calculated from 1 kb windows across the mitochondrial genomes of *D. flaviventris* Dfl03 (**a**) and Dfl04 (**b**), and *D. suweonensis* Dsu04 (**c**) and Dsu08 (**d**). The red bars above the peaks indicate putative repeats identified by the Tandem Repeats Finder. The dashed red lines represent the threshold value (0.0025), determined by the unsupervised method. The peaks near 0 correspond to non-repetitive regions, while the peaks around 0.006 indicate a limited number of repetitive windows. Zoomed-in views of the two major peak regions for each dataset of *D. flaviventris* Dfl03 (**e**) and Dfl04 (**f**), *D. suweonensis* Dsu04 (**g**) and Dsu08 (**h**).

Repeat detection using D2RreadFilter software revealed that repetitive sequences were concentrated exclusively in the D-loop region in both *D. flaviventris* and *D. suweonensis* (Figure 4). Peaks near 0.006 indicate repetitive regions, while those near 0 correspond to non-repetitive windows. The threshold (0.0025) effectively distinguished these patterns. The D-loop regionis known to play a crucial role in the regulation of mtDNA replication and transcription [19]. If the level of genetic variation is high, tandem repeats often exhibit a high degree of heterozygosity and a multi-allelic nature [20]. In *D. flaviventris* and *D. suweonensis*, tandem repeats were found only in this region (Table 2), with repeat units ranging from 121 to 244 bp, 3.3 to 10.5 copies, and 94–100% matches.

**Figure 5:**
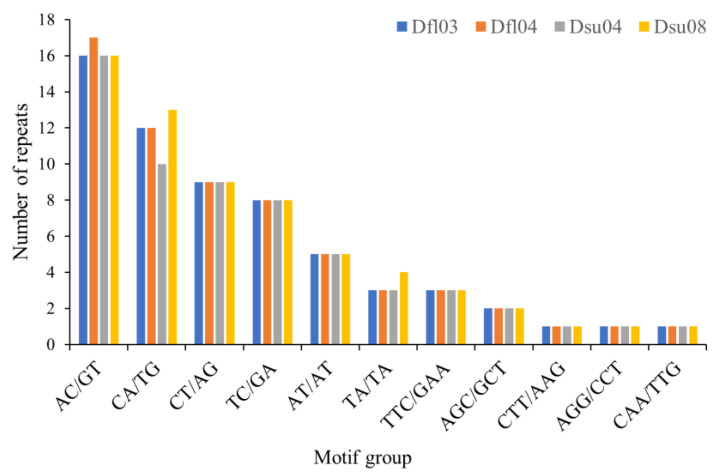
Distribution of microsatellite motif groups in mitogenomes of two *Dryophytes* species (Dfl: *D. flaviventris*; Dsu: *D. suweonensis*).

The distribution of microsatellite motif groups in the mitogenomes of *D. flaviventris* and *D. suweonensis* showed a predominance of dinucleotide motifs (AC/GT, CA/TG, and CT/AG) across all the samples (Figure 5). Among them, AC/GT and CA/TG were the most abundant. Trinucleotide motifs were less frequent, with TTC/GAA being the most common. Tetranucleotide to hexanucleotide motifs were absent in all the samples. The consistency in motif patterns suggests a conserved microsatellite distribution among these species.

No interspersed repeats were detected by RepeatMasker in any of the samples.

### 2.6. Synonymous and Nonsynonymous Substitution Rate

The average Ka (nonsynonymous)/Ks (synonymous) ratio for the PCGs was less than 1 (0.007–0.901), indicating purifying selection. Furthermore, no difference was found in the Ka/Ks ratio values between the two species (D = 0, *p*-value = 1). In the *Nd6* gene, the Ka/Ks ratio was greater than 1. Except for *Nd6*, the remaining PCGs exhibited purifying selection, indicating that they evolved to eliminate deleterious mutations [21]. Notably, in *Nd6*, the Ka/Ks values were above 1, indicating positive selection, which suggests an evolution toward retaining beneficial mutations. Specifically, *Nd6* in both *D. flaviventris* and *D. suweonensis* shows the same evolutionary trend. The positive selection on *Nd6* in *D. flaviventris* and *D. suweonensis* may be associated with adaptation to cold stress. Unlike other frogs, these species survive winters in wetlands, suggesting they may have undergone positive selection to enhance cold tolerance. Studies have shown that the positive selection of mitochondrial genes can occur as an adaptive response to low temperatures [22,23]. Given that the Republic of Korea experiences four distinct seasons, it is possible that specific regions of the PCGs in both species have undergone positive selection to support winter survival.

### 2.7. Phylogenetic Analysis

The phylogenetic tree based on maximum likelihood (ML) and Bayesian inference (BI) analysis showed that *D. flaviventris* and *D. suweonensis* were grouped into a single lineage, with strong support. This single lineage suggests minimal differences in the mitogenome sequences between the two species, indicating that they may be the same species. The genetic distance between them is also very small. Additionally, it was found that *Hyla tsinlingensis* (KP212702) was misidentified in a previous study and should actually be classified as *D. immaculatus*. Furthermore, *D. suweonensis* and *D. immaculatus* have been identified as synonymous [13].

**Figure 6:**
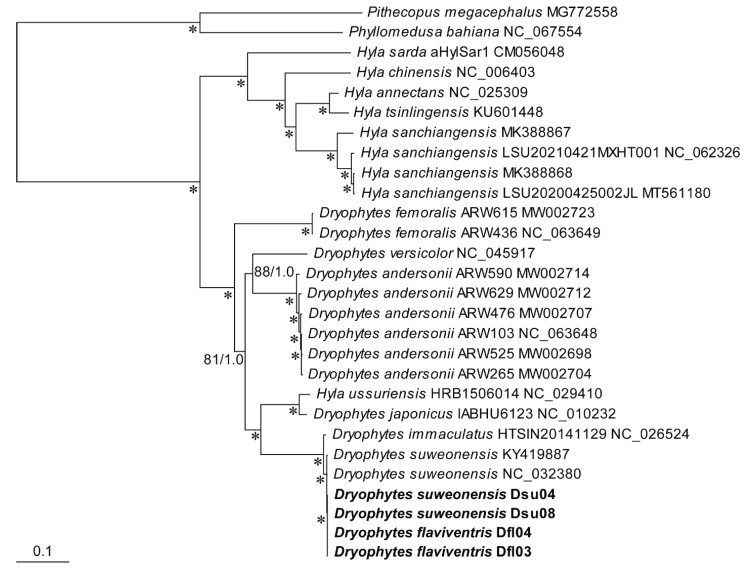
A maximum likelihood (ML) and Bayesian inference (BI) tree based on all the protein-coding genes of the complete mitogenomes of each specimen of the two treefrog species—*Dryophytes flaviventris* Dfl03 and Dfl04, and *D. suweonensis*—Dsu04 and Dsu08. The numeric values at the nodes are ML bootstraps (above 50%)/Bayesian posterior probabilities. The newly assembled mitogenomes of the four treefrogs is highlighted in the bold. Two species, *Phyllomedusa bahiana* (NC_067554) and *Pithecopus megacephalus* (MG772558), were chosen as outgroups. The asterisks (*) indicate clades with a strong ML (100% bootstraps) and Bayesian (1.0 posterior probabilities) nodal support.

The phylogenetic tree based on all the PCGs of the complete mitogenomes of all *Hyla* and *Dryophytes* species showed that they formed a monophyletic group supported by the highest bootstrap value and Bayesian posterior probability in ML and BI analyses, respectively, with respect to the two outgroup species (Figure 6). They further bifurcated mainly according to the generic taxonomy, except for *H. ussuriensis,* that emerged among *Dryophytes* species. *Dryophytes* species and *H. ussuriensis* further emerged according to their species taxonomy, except for *D. flaviventris* and *D. suweonensis,* that showed the closest relationship to *D. immaculatus*. The two specimens of *D. flaviventris* and four specimens of *D. suweonensis* clustered together, but were phylogenetically not distinguishable.

### 2.8. Characteristic Analysis of Comparative Genome

The difference in genome size among closely related species reflects their evolutionary processes and is linked to species divergence [18,19]. Intraspecific variations have also been reported and are sometimes associated with environmental conditions, suggesting that genome size is a selective trait related to cell and body size [24]. Therefore, we utilized comparative genomics to identify genetic similarities and differences between *D. flaviventris* and *D. suweonensis*. Comparative genomics involves analyzing the mtDNA sequences of organisms to construct phylogenetic trees that depict evolutionary relationships.

Analyses using ddRAD in *D. flaviventris* have shown differences from *D. suweonensis* [10], while all evidence indicated that the maternal lineages of the two species were perfectly matched in our study. There are some problems with using ddRAD to select single-nucleotide polymorphisms (SNPs). Since ddRAD only analyzes specific regions of the genome [25,26], it can introduce coverage bias in certain genomic regions [27,28], which may distort the analysis results. Also, identifying their phylogeny, the two species appeared to be very close, but in closely related species, SNPs vary less frequently, which could make it difficult to distinguish true variation from error.

The mitogenome arrangement was found to be the same between *D. flaviventris* and *D. suweonensis*. Also, there were no differences in tRNA gene arrangements. When comparing the nucleotide sequences, there were no observed differences in the sequence lengths and/or structures of the genes. AT/GC-skews and phylogenetic trees were found to be not likely to be different between the two species. Generally, mitochondrial sequence differences between them exhibit clear differences in length [29,30,31]. However, their genetic differences in mitogenome size, AT/GC-skews, and phylogenetic trees can be explained through two hypotheses.

First, due to the maternally inherited nature of mitogenomes, the mitochondrial sequences of *D. flaviventris* and *D. suweonensis* may have coincidentally aligned due to hybridization. This could be an example of mitochondrial capture, defined as mitogenome introgression, where the mitochondrial genome of one species is replaced by that of another species through selective backcrossing with one of the parent species [32,33]. Therefore, studies using nuclear DNA would be required for the definitive identification of *Dryophytes* species.

Second, it is possible that *D. flaviventris* and *D. suweonensis* are actually synonymous. Genetic similarities in mitochondrial sequences have led to reports of synonymy in other species as well [34]. The mitogenome verification conducted in this study makes the first hypothesis highly unlikely. Several pieces of evidence support the second hypothesis. For instance, the two speices have been reported to exhibit no differences in their calls [35]. The species were previously distinguished based on morphological differences, specifically the fact that the webbing between the second and third toes of *D. flaviventris* is smaller than that of *D. suweonensis* [10]. However, this morphological difference is not considered a reliable basis for accurate classification. Therefore, it is believed that the two species are genetically very similar and likely conspecific.

Molecular tools have become essential in conservation-based research [36], providing information on population biology and species-level relationships [37,38]. As a result, molecular systematics plays a crucial role in identifying and conserving endangered species [39,40]. While *D. suweonensis* is already protected as an endangered species, if *D. flaviventris* is recognized as a distinct species, its conservation would become urgent due to its narrower distribution range compared to *D. suweonensis*.

In the future, we will conduct studies on the nuclear DNA of both species to test the first hypothesis of mitogenome congruence due to hybridization and to confirm bi-parental inheritance. Through these analyses, we aim to establish a clearer phylogenetic understanding of tree frogs in the Republic of Korea.

## 3. Materials and Methods

### 3.1. Sample Collection and Genomic DNA Extraction

Two *D. flaviventris* specimens were captured in 2021, one from Wanju-gun, Jeollabuk-do, and the other from Buyeo-gun, Chuncheongnam-do, the Republic of Korea. These two samples were preserved in absolute ethanol and deposited in the archives of the Conservation Genome Resource Bank for Korean Wildlife (CGRB) (http://www.cgrb.org/, accessed on 19 July 2022). Two samples of *D. suweonensis* were collected from Chungju city and Iksan city in April–May 2022. 

A piece of tissue was excised for genomic DNA (gDNA) extraction using an APrep™ gDNA Tissue Kit (APBIO Co., Namyangju, the Republic of Korea) following the manufacturer’s instructions. The qualification of the extracted gDNA was performed using a NanoDrop One Microvolume UV-Vis Spectrophotometer (Thermo Fisher Scientific Inc., Wilmington, DE, USA). 

### 3.2. Primer Design and Long-Range PCR

A total of 47 complete mitogenome sequences belonging to the family Hylidae were retrieved from the GenBank database in the NCBI (https://www.ncbi.nlm.nih.gov/, accessed on 6 February 2023) and aligned with ClustalW in Bioedit v.7.2 [41]. Alignments were visually inspected and confirmed; any ambiguous alignments were manually corrected. Two sets of primers were newly designed in this study, based on the matrix, to amplify the entire mitogenome in overlapping fragments for the four treefrog specimens (Table 3). Each 20 μL long-range PCR for each primer set was amplified with a proofreading and ultrahigh fidelity DNA polymerase was ensured using an Invitrogen™ Platinum™ SuperFi™ PCR kit (Thermo Fisher Scientific Inc.). The amplification was carried out in the ProFlex™ PCR System (Thermo Fisher Scientific Inc.). The reactions were run under the following conditions: one initial cycle at 30 s at 98 °C, followed by 35 cycles of 10 s of denaturing at 98 °C, 10 s of annealing at 60 °C, a 5 min extension at 72 °C, and then finalized by one cycle of 5 min elongation at 72 °C. All the PCR products were visualized on a 1.5% agarose gel to verify amplification success.

### 3.3. Library Preparation and Sequencing

The successfully amplified PCR products were purified using an AccuPrep^®^ PCR/Gel Purification Kit (Bioneer, Daejeon, the Republic of Korea) and qualified using Quant-iT™ PicoGreen™ dsDNA Assay Kits (Thermo Fisher Scientific, Inc.). PCR amplicons of overlapping fragments (total maximum concentration of 200 ng) of each species were pooled together and prepared for library preparation using Illumina DNA Prep kits (Illumina Inc., San Diego, CA, USA). After quantification using Quant-iT™ PicoGreen™ dsDNA Assay Kits (Thermo Fisher Scientific Inc.), the library products were sequenced with the Illumina iSeq100 System (Illumina) according to the manufacturer’s instructions utilizing an Illumina iSeq™ 100 i1 Reagent v2 kit (Illumina). The sequences were sorted into FASTQ files, and the adaptor and index sequences were removed using the onboard Illumina FASTQ workflow.

### 3.4. Mitochondrial Genome Assembly

Raw FASTQ files were assessed using FastQC v.0.12.1 [42]. Subsequently, they were imported to Geneious Prime v.2023.2.1 [43] for quality control and assembly. The raw reads were pre-processed by pair-end merging, removing duplicates, discarding reads shorter than 50 bp, and trimming low-quality ends using the BBDuk trimmer at default settings from the BBtools plugin version 38.90 [44] in Geneious Prime version 2021.1.1. The trimmed reads were assembled with the ‘map to reference’ approach in Geneious Prime using a validated mitogenome of *D. suweonensis* (GenBank accession number KY419887) at default setting. Consensus sequences at the highest 60% identical threshold were manually checked and subjected to variant calling using the ‘Variant/SNP finder’ option at default settings. A table of all the variant annotations was obtained. Only variants which were lower than the maximum variant *p*-value (0.0001%) and greater than 50% of the variant frequency were selected to be applied to the consensus sequence.

### 3.5. Mitochondrial Gene Annotation and Codon Usage Analysis

The assembled consensus sequences obtained from the variant calling process were visualized and annotated in Geneious Prime. The identifications of the exact start and stop codons of all the protein-coding genes (PCGs) and the boundaries of two ribosomal RNA (rRNA) genes were carried out after aligning the newly analyzed complete mitochondrial sequences in this study and other sequences of the family Hylidae. Transfer RNA (tRNA) genes were identified by tRNAscan-SE 1.21, employing the vertebrate mitochondrial genetic code [45]. The boundaries of all the genes were inferred by the flanking genes under the assumption that there were neither intergenic spacers nor overlaps, and we then performed repeated ‘map to reference’ operations using Geneious Prime with default settings. The complete mitogenome sequence of *D. suweonensis* (KY419887) was used as a reference sequence for the four treefrog specimens newly analyzed in this study. The schemes of the circular mitogenome were constructed with the GenomeVx Web Browser, http://wolfe.ucd.ie/GenomeVx/ (accessed on 6 April 2023) [46]. The relative synonymous codon usage (RSCU) was calculated by MEGA X version 10.0 [47]. The four newly sequenced mitogenomes of the *Dryophytes* species were submitted to GenBank with the accession numbers PQ490412–PQ490415.

### 3.6. Repeat Sequence Analysis

Repetitive regions within the two *Dryophytes* species mitogenomes were identified using using D2RReadFilter software (https://github.com/chansigit/D2R_codes, accessed on 4 February 2025) with default parameters [48], and the threshold value was determined using the unsupervised method. Simple sequence repeats (SSRs) were detected using GMATA v.2.3 [49]. Tandem repeats with repeat units >6 bp were identified using Tandem Repeats Finder v.4.09 [50] with default parameters. Interspersed repeats were identified using RepeatMasker v.4.1.7 with default settings [51].

### 3.7. Synonymous and Nonsynonymous Substitution Rate

The Ka (nonsynonymous)/Ks (synonymous) ratio is an important value for understanding molecular evolutionary dynamics [52]. A Ka/Ks = 1 indicates neutral mutation, Ka/Ks > 1 indicates positive selection, and Ka/Ks < 1 indicates negative (purifying) selection. We used 13 PCGs to obtain Ka/Ks ratios. Their Ka/Ks ratios in *D. flaviventris*, *D. suweonensis*, and *D. japonicus* were calculated using DnaSP v5 [53]. To test the statistical significance of the difference in the Ka/Ks ratio between *D. flaviventris* and *D. suweonensis*, the Kolmogorov–Smirnov test was used to calculate the p-value, allowing for direct comparisons [54].

### 3.8. Phylogenetic Analysis

The concatenated nucleotide matrix of 27 mitogenome sequences of frog species belonging to the family Hylidae retrieved from the GenBank database and those of the four treefrog specimens in this study were aligned and manually refined using the ClustalW version 1.4 multiple alignment function of BioEdit version 5.0.9 with default parameters for constructing a phylogenetic tree. All 13 PCGs from the matrix were extracted and employed in building a maximum likelihood (ML) tree and executing the Bayesian inference (BI) analysis. The GTRGAMMAI model, a general time-reverse (GTR) model incorporating invariant sites and a gamma distribution, was selected as the optimal phylogenetic model by JModelTest v. 2 [55]. The ML analysis was performed with RAxML 7.0.4 using 1000 nonparametric bootstrap inferences [56,57]. For BI analysis, MrBayes v. 3.1.2 was utilized, employing four independent Markov chains with 1,000,000 generations and discarding the initial 25% as burn-in [58]. The resulting tree was visualized using TreeViewX v. 0.5.0 [59]. Two species, *Phyllomedusa bahiana* (NC_067554) and *Pithecopus megacephalus* (MG772558), were chosen as outgroups.

## 4. Conclusions

In this study, we sequenced and characterized the complete mitogenomes of *D. flaviventris* for the first time, as well as *D. suweonensis* in the Republic of Korea, revealing small differences (0–7 bp) in their mitogenome sizes. No variations were observed in gene rearrangements or tRNA structures, and both repeat sequence analysis in the D-loop region and RSCU analysis showed no distinct differences. Phylogenetic analysis confirmed that the two tree frog species form a monophyletic group. Despite conducting a detailed mitogenome analysis, we found no genetic differences between the two species, strongly suggesting their conspecificity. To further investigate this hypothesis, we will conduct nuclear DNA studies on both species to test whether their mitogenome congruence is due to hybridization.

## Figures and Tables

**Table 1:** AT/GC-skews of 13 protein-coding genes (PCGs), rRNAs, tRNAs, and D-loop region.

	Species ID		Dfl03	Dfl04	Dsu04	Dsu08	Dj (AB303949)
Nucleotide frequency (%)	Whole	A	29.12	29.13	29.14	29.14	29.59
		G	14.93	14.92	14.92	14.91	14.50
		T	28.48	28.50	28.48	28.49	30.50
		C	27.47	27.44	27.47	27.46	25.41
		A + T	57.60	57.64	57.61	57.63	60.09
		AT-skew	0.01	0.01	0.01	0.01	−0.02
		GC-skew	−0.30	−0.30	−0.30	−0.30	−0.27
	PCGs	A	27.30	27.32	27.30	27.31	28.40
		G	14.07	14.05	14.07	14.06	13.45
		T	29.18	29.20	29.19	29.20	31.12
		C	29.45	29.43	29.43	29.43	27.03
		A + T	56.48	56.52	56.49	56.51	59.52
		AT-skew	−0.03	−0.03	−0.03	−0.03	−0.05
		GC-skew	−0.35	−0.35	−0.35	−0.35	−0.34
	rRNAs	A	33.78	33.78	33.78	33.77	34.89
		G	18.55	18.55	18.55	18.54	17.88
		T	25.06	25.02	25.06	25.05	25.53
		C	22.61	22.65	22.61	22.64	21.70
		A + T	58.84	58.80	58.84	58.82	60.42
		AT-skew	0.15	0.15	0.15	0.15	0.15
		GC-skew	−0.10	−0.10	−0.10	−0.10	−0.10
	tRNAs	A	31.74	31.74	31.83	31.74	31.91
		G	17.76	17.76	17.68	17.76	17.68
		T	26.56	26.56	26.56	26.56	26.97
		C	23.93	23.93	23.93	23.93	23.44
		A + T	58.31	58.31	58.39	58.31	58.88
		AT-skew	0.09	0.09	0.09	0.09	0.08
		GC-skew	−0.15	−0.15	−0.15	−0.15	−0.14
	D-loop	A	30.41	30.42	30.48	30.53	28.56
		G	13.95	13.96	13.89	13.87	14.28
		T	29.57	29.7	29.54	29.59	33.12
		C	26.06	25.92	26.09	26.02	24.03
		A + T	59.98	60.13	60.02	60.11	61.69
		AT-skew	0.01	0.01	0.02	0.02	−0.07
		GC-skew	−0.30	−0.30	−0.31	−0.30	−0.25

**Table 2:** The distribution of tandem repeats in the mitogenomes of *Dryophytes flaviventris* Dfl03and Dfl04, and *D. suweonensis* Dsu04 and Dsu08. T01-T03 indicate different occurrences of tandem repeats.

Sample	Repeat Name	Size	No. Copy	Matches (%)	Start	End	Location
Dfl03	Dfl03-T01	160	3.3	99	15,439	15,961	Putative control region
	Dfl03-T02	123	10.5	95	17,336	18,615	Putative control region
	Dfl03-T03	242	5.2	94	17,336	18,615	Putative control region
Dfl04	Dfl04-T01	160	3.3	99	15,439	15,961	Putative control region
	Dfl04-T02	121	10.5	95	17,335	18,614	Putative control region
	Dfl04-T03	244	5.2	96	17,335	18,614	Putative control region
Dsu04	Dsu04-T01	160	3.3	100	15,439	15,961	Putative control region
	Dsu04-T02	121	10.5	95	17,335	18,608	Putative control region
	Dsu04-T03	242	5.2	95	17,335	18,608	Putative control region
Dsu08	Dsu08-T01	160	3.3	99	15,440	15,962	Putative control region
	Dsu08-T02	123	10.5	95	17,337	18,614	Putative control region
	Dsu08-T03	244	5.2	94	17,337	18,614	Putative control region

**Table 3:** The two sets of PCR primers newly designed in this study to amplify the entire mitogenomes in overlapping fragments for the two tree frog species, *Dryophytes flaviventris* and *D. suweonensis*.

Primer	Oligonucleotide (5′ → 3′)	Expected Amplicon Size (bp)
Set 1		
HYL-MT-02336f	AGACGAGAAGACCCTDTGGA	ca. 7700
HYL-MT-10028r	TRAGYCGAAATCAGCTGTCTTT	
Set 2		
HYL-MT-07929f	AGCGACAGCCTTTTAAGCT	ca. 13,400 bp
HYL-MT-02706r	TGATCTGAGTTCAGACCGGA	

## Data Availability

All DNA data are available in the NCBI: PQ490412–PQ490415. Please contact the corresponding author (kskim@nie.re.kr) to request the original DNA reads. https://www.ncbi.nlm.nih.gov/nuccore/PQ490412.1/ (accessed on 27 October 2024). https://www.ncbi.nlm.nih.gov/nuccore/PQ490413.1/ (accessed on 27 October 2024). https://www.ncbi.nlm.nih.gov/nuccore/PQ490414.1/ (accessed on 27 October 2024). https://www.ncbi.nlm.nih.gov/nuccore/PQ490415.1/ (accessed on 27 October 2024).

## Supplementary Materials

The following supporting information can be downloaded at: https://www.mdpi.com/article/10.3390/ijms26062423/s1.
