# Supplementary material for: First Complete Mitochondrial Genome Analysis of Tree Frog, Dryophytes flaviventris and Comparison with Dryophytes suweonensis
Source: Int J Mol Sci. 2025 Mar 7;26(6):2423. doi: 10.3390/ijms26062423 (PMC11942598; doi:10.3390/ijms26062423)
Supplement: Supplementary file 1 [file ijms-26-02423-s001.zip › ijms-3485777-supplementary.pdf]

First Complete Mitochondrial Genome Analysis of Tree Frog, *Dryophytes flaviventris* and Comparison with *Dryophytes suweonensis*

Nakyung Yoo [1,†], Kang-Rae Kim [2,†], Biet Thanh Tran [3], Keun-Yong Kim [3], Mi-Sook Min [4], Ju-Duk Yoon [1,*] and Keun-Sik Kim [1,*]

[1] Restoration Research Team (Fishes/Amphibians & Reptile), Research Center for Endangered Species, National Institute of Ecology, Yeongyang 36531, Republic of Korea; nkyoo96@nie.re.kr

[2] Southeast Sea Fisheries Research Institute, National Institute of Fisheries Science, Namhae 53085, Republic of Korea; kimkangrae9586@gmail.com

[3] Genetic Analysis Team, AquaGenTech Co., Ltd., Busan 48228, Republic of Korea; aquagentech@naver.com (B.T.T.); koby0323@hanmail.net (K.-Y.K.)

[4] Conservation Genome Resource Bank for Korean Wildlife (CGRB), BK21 Program for Veterinary Science, College of Veterinary Medicine, Seoul National University, Seoul 08826, Republic of Korea; minbio@yahoo.co.kr

* Correspondence: grandblue@nie.re.kr (J.-D.Y.); kskim@nie.re.kr (K.-S.K.); Tel.: +82-54-680-7361 (J.-D.Y.); +82-54-680-7362 (K.-S.K.)

† These authors contributed equally to this work.

Supplementary

Table S1. Summary of long-range PCR sequencing and data processing for *Dryophytes flaviventris* (Dfl03 and Dfl04) and *D. suweonensis* (Dsu03 and Dsu04) in Korea.

Sample	Data	Total bases (bp)	Number of reads	GC (%)	Q20 (%)	Q30 (%)
Dfl03	Raw	114,830,069	899,918	44.0	97.2	94.1
	Preprocessing	84,000,629	687,020	43.5	100.0	99.0
Dfl04	Raw	147,646,043	1,180,464	43.4	97.0	93.8
	Preprocessing	103,430,090	849,096	42.8	100.0	99.0
Dsu04	Raw	122,728,113	967,424	43.3	97.1	94.0
	Preprocessing	87,668,798	716,222	42.8	100.0	99.0
Dsu08	Raw	154,962,717	1,271,642	43.6	96.9	93.8
	Preprocessing	107,569,991	898,832	43.1	100.0	99.0

Table S2. Mapping results of *Dryophytes flaviventris* (Dfl03 and Dfl04) and *D. suweonensis* (Dsu03 and Dsu04) mitochondrial genomes.

Sample	Mapping reads (read)	Base coverage (bp)	Coverage (%)	Min depth	mean depth	Max depth
Dfl03	547,223	18,617	100	36	3,771	13,358
Dfl04	788,281	18,616	100	188	5,262	17,303
Dsu04	680,322	18,610	100	9	4,585	14,952
Dsu08	813,492	18,616	100	157	5,382	17,899

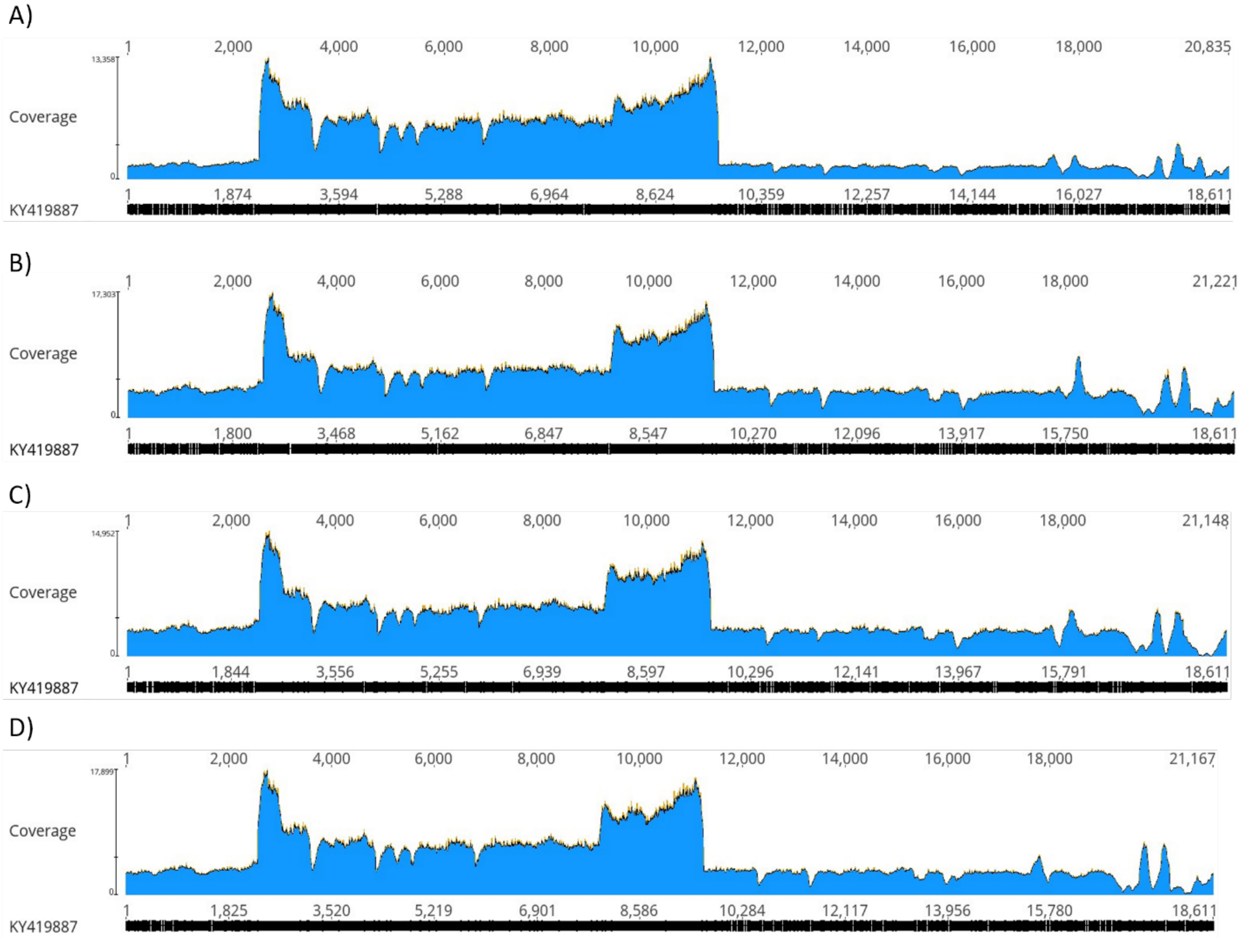

Figure S1. Coverage depth of *Dryophytes flaviventris* (A: Dfl03 and B: Dfl04) and *D. suweonensis* (C: Dsu03 and D: Dsu04) mapped to KY419887. The black bar represents the reference genome annotation. The x-axis shows nucleotide positions, and the y-axis shows read depth.
